# Nanomaterials for Combined Stabilisation and Deacidification of Cellulosic Materials—The Case of Iron-Tannate Dyed Cotton

**DOI:** 10.3390/nano10050900

**Published:** 2020-05-08

**Authors:** Nicoletta Palladino, Marei Hacke, Giovanna Poggi, Oleksandr Nechyporchuk, Krzysztof Kolman, Qingmeng Xu, Michael Persson, Rodorico Giorgi, Krister Holmberg, Piero Baglioni, Romain Bordes

**Affiliations:** 1 Swedish National Heritage Board, Heritage Science, 62122 Visby, Sweden; nicpal@kth.se; 2 Department of Chemistry and CSGI, University of Florence, 50019 Sesto Fiorentino (Florence), Italy; xu@csgi.unifi.it (Q.X.); giorgi@csgi.unifi.it (R.G.); baglioni@csgi.unifi.it (P.B.); 3 Department of Chemistry and Chemical Engineering, Chalmers University of Technology, 41296 Gothenburg, Sweden; oleksandr.nechyporchuk@ri.se (O.N.); krzysztof.kolman@nouryon.com (K.K.); michael.persson@nouryon.com (M.P.); krister.holmberg@chalmers.se (K.H.); 4 Nouryon, 44534 Bohus, Sweden

**Keywords:** stabilisation, deacidification, nanoparticle, paper, canvas, iron-tannate dye, acid-catalysed degradation

## Abstract

The conservation of textiles is a challenge due to the often fast degradation that results from the acidity combined with a complex structure that requires remediation actions to be conducted at several length scales. Nanomaterials have lately been used for various purposes in the conservation of cultural heritage. The advantage with these materials is their high efficiency combined with a great control. Here, we provide an overview of the latest developments in terms of nanomaterials-based alternatives, namely inorganic nanoparticles and nanocellulose, to conventional methods for the strengthening and deacidification of cellulose-based materials. Then, using the case of iron-tannate dyed cotton, we show that conservation can only be addressed if the mechanical strengthening is preceded by a deacidification step. We used CaCO_3_ nanoparticles to neutralize the acidity, while the stabilisation was addressed by a combination of nanocellulose, and silica nanoparticles, to truly tackle the complexity of the hierarchical nature of cotton textiles. Silica nanoparticles enabled strengthening at the fibre scale by covering the fibre surface, while the nanocellulose acted at bigger length scales. The evaluation of the applied treatments, before and after an accelerated ageing, was assessed by tensile testing, the fibre structure by SEM and the apparent colour changes by colourimetric measurements.

## 1. Introduction

Cellulosic materials have historically been widely used for documentation, household items as well as for artistic purposes, in the form of paper, carton, canvas, or textiles, and by that have become a ubiquitous material, which conservators have to deal with. Cellulose is a natural polymer that is essential for the mechanical properties of plants, from which it is extracted; it is built on the repeating unit glucose linked through β-1,4-glycosidic bonds [1]. The degree of polymerisation (DP) of cellulose, which typically ranges from 9000 to 15,000 [2], is one of the key indexes to assess the condition of cellulosic-based materials. Even though cellulose is a relatively stable polymer, it is known to undergo degradation and the most important degradation mechanism is acid-catalysed hydrolysis of the β-1,4-glycosidic bond [3,4,5], resulting in a decrease of the DP and, consequently, a decrease of the mechanical resistance of the material. New insights into the changes in the macromolecular network of cellulose after acid-catalysed hydrolysis were obtained using NMR diffusometry and relaxometry [6,7]. The degradation of cellulose-based material, which may take place at room temperature, is due to environmental and endogenous factors. When cellulosic materials are used in relation to artefacts, the endogenous factors may originate from the making process or from a subsequent treatment. Most of the compounds are acidic or may develop acidic compounds upon ageing. For instance, alum and rosin, traditionally used for sizing of paper, are responsible for the bad conservation status of paper produced from the beginning of the 19th until the mid 20th century. Paints and varnishes based on drying-oil resins are acidic or may develop acidic compounds upon natural ageing, posing a threat to linen and cotton canvas [8,9]. In the case of archaeological and waterlogged wood, many consolidants used in the past are known to be acidic or to release acidic compounds upon ageing, thus promoting further degradation [10]. Scientists and conservators have recently put increasing attention to the conspicuous number of documentary, as well as historical and artistic, cellulosic materials in need of urgent maintenance [11,12,13,14,15].

Considering the detrimental impact of acidic compounds in the degradation of cellulosic materials, deacidification, i.e., the neutralisation of acidity in the material, is a key step in the remediation process that secures the preservation of these heritage materials. In most of the cases, reinforcement is also needed, which is usually devoted to stabilisation of the mechanical properties so as to allow manipulation and handling of the artefact. Depending on the original material, different approaches can be used, such as relining for canvases [16] or mending using Japanese paper and adhesives for paper [17].

In recent years, solutions based on nanomaterials were introduced in the field of cultural heritage conservation [18,19]. These innovative systems are often more efficient and more compatible with the original artefacts than traditional methods and have a broad applicability. Over the years, several methods have been proposed for the deacidification of cellulose-based materials [20,21], with strong emphasis on paper. The work on textiles has been much more limited, even though one may find some examples about the neutralisation of naturally aged, acidic linen, cotton and flax canvas [9,22,23]. Aqueous solutions of calcium (or magnesium) bicarbonate and calcium hydroxide are traditionally used for the deacidification of paper, and the deacidification is carried out via immersion of paper sheets separated from the binder [24]. Despite being effective, especially on single paper sheets, there are severe drawbacks associated with the use of this method: (i) oxidised cellulose displays a high sensitivity to strongly alkaline environments; (ii) inks and sizing agents can be solubilised or altered when immersed in an alkaline aqueous solutions [25,26,27,28,29].

The removal or alteration of the original components of the artefacts during a deacidification treatment can be avoided using solvents less polar than water [28]. Deacidification treatments based on the use of such solvents are called “non-aqueous methods” and are presently preferred over traditional aqueous methods [21,30,31,32,33,34]. The use of alkaline nanoparticles, mainly calcium or magnesium hydroxide, dispersed in short-chain alcohols for the deacidification of paper and canvas was proposed in the early 2000s [35,36]. Since then, several synthetic strategies and formulations of nanoparticles in different carrier solvents were tested.

Over the years, alkaline nanoparticles have been successfully used on waterlogged wood [37,38,39], on wood from organ pipes [40], and on paper and canvas [35,36,41,42,43]. The use of alkaline nanoparticles underwent extensive assessment with positive results [28,29,44,45,46,47]. Research efforts have continued to increase applicability of such particles with, for instance, the use of apolar solvents [48] which allows for a safer intervention even on creased and folder paper.

The second challenge in the stabilization of fibrous materials is to recover the loss mechanical strength, as manifested by a reduction of maximum force and elongation at break, in a material where chemical bonds were irreversibly broken, and which is continuously subjected to stress, due to the continuous variation of temperature and humidity. 

The hierarchical structure of fibrous-based materials imposes to treat and act at several length scales. This is an aspect where the conventional approach, which consists of strengthening degraded fibrous materials with a macroscopic treatment, fails. Typically, for mildly degraded materials, treatments with compatible solutions of cellulose derivative are chosen, while for more severely degraded materials, a non-degraded support is attached. For instance, Japanese paper is usually applied by means of a cellulose-based glue to treat paper artefact [49], whereas, in the case of canvas, a new pristine canvas is applied on the back of the artwork. This operation is called *lining* or *re-lining*. The adhesive can either be natural (such as wax-resin or glue-paste), or synthetic (acrylate and methacrylate polymers, e.g., Plextol B500, D360 or BEVA 371) [50,51,52]. Water-based adhesives are less commonly employed because water permeating the canvas may induce dimensional changes (swelling, shrinkage) that would, in turn, stress the paint layer. However, the recent trends, which are in line with the minimal intervention philosophy, raised an interest from the community to explore the possibility of using nanomaterials, with the ambition of mitigating some of the shortcomings of lining, such as the large weight addition and the loss of the original appearance [53].

Nanocellulose, (i.e., cellulose nanocrystals, cellulose nanofibres, also referred to as nanofibrillated cellulose, and bacterial cellulose), and inorganic nanoparticles have lately been tested to this end. Cellulose nanocrystals and cellulose nanofibres proved to be the most efficient as they tend to accumulate on the surface of the textiles or paper with limited penetration, while at the same time being transparent and acting as a micro- or nanolining [54,55,56,57,58]. Nanocellulose can also be combined with soluble cellulose derivatives, such as methyl cellulose. 

The use of inorganic nanoparticles was also explored as they act at much shorter length scales and their efficiency depends strongly on the degree of coverage of the fibres. Silica nanoparticles proved to be particularly efficient. The main effect observed in term of strengthening is an increase in the fibre stiffness [59], most likely owing to a dense packing of the particles at the fibre surface. Inorganic nanoparticles may not be fully compatible with cellulose-based fibres, and that is why surface modification with a soluble cellulose derivative, e.g., carboxymethyl cellulose can be conducted. This type of surface treatment proved to greatly enhance the control of the nanoparticle deposition while mitigating the effect of aggregation and undesired accumulation of the nanoparticles [59]. 

Interestingly, synergistic effects can be obtained when using nanocellulose and silica nanoparticles, and the increase of the fibres’ stiffness due to the inorganic nanoparticles can be compensated for by the structural reinforcement provided by the nanocellulose nanolining [60]. 

There are only few examples of combined deacidification and stabilisation treatments reported in the literature, and these are exclusively designed to address the conservation of paper and manuscripts [46,61,62,63,64]. Herein, we selected iron-tannate textiles as representatives of strongly degraded fibrous materials in need for a stabilising conservation. The problem of iron-tannate dyes is symptomatic of a wide variety of textiles collections, such as Maori piu-piu, Kuba cloth from Congo, tapa of the Pacific, Japanese hina dolls, 17th century Dutch clothing and many more.

The treatment chosen was developed within the European project NANORESTART and consisted, for the deacidification part, of nanoparticles of calcium carbonate treated with carboxymethyl cellulose (CMC) to grant compatibility with cellulose substrate, while the mechanical stabilization was achieved using coated silica nanoparticles and nanocellulose.

## 2. Materials and Methods

### 2.1. Materials

A dispersion of silica nanoparticles (SNP), (Levasil CS50-28, former Bindzil 50/80), was supplied by Nouryon (formerly AkzoNobel Pulp and Performance Chemicals, Sweden), Bohus, Sweden. The surface area of these particles was 80 m^2^/g which corresponds to a spherical diameter of 35 nm. The suspension had a pH of 9 (sodium ion stabilised), a concentration of 50 wt% silica, and a density of 1.4 g/mL.

Branched polyethylenimine (PEI) with an average molecular weight (Mw) of 25,000 g/mol was purchased from Sigma-Aldrich (Steinheim, Germany). Sodium carboxymethylcellulose (CMC) supplied by Nouryon (Bohus, Sweden) had a degree of substitution of 0.77 and a viscosity of 12 mPa·s (at 1%). The Mw of the CMC was 650,000 g/mol (determined by size exclusion chromatography). Polyvinylpyrrolidone (PVP) with a Mw of 10,000 g/mol was purchased from Sigma-Aldrich (Steinheim, Germany). The SNP formulation is reported elsewhere [59,60].

Cellulose nanofibres (CNF) in the form of an aqueous suspension was kindly provided by Stora Enso AB (Karlstad, Sweden). The CNF was produced from softwood pulp (ca. 75% pine and 25% spruce, containing 85% cellulose, 15% hemicellulose, and traces of lignin, as reported by the supplier). Cellulose nanocrystals (CNC) in powder form was purchased from CelluForce (Windsor, QC, Canada). It was produced from bleached kraft pulp by sulfuric acid hydrolysis. Ethanol (99.5%, Sigma-Aldrich, Steinheim, Germany), metal granular calcium (99%, Aldrich, Steinheim, Germany), and diethyl carbonate (99%, Aldrich, Steinheim, Germany) were used for the preparation of the CaCO_3_ nanoparticle dispersion, following a procedure reported elsewhere [39,65]. Isopropanol, used for dilution of selected treatments, was supplied by VWR Chemicals (98%, Radnor, PA, USA).

### 2.2. Sample Preparation

**Figure 1:**
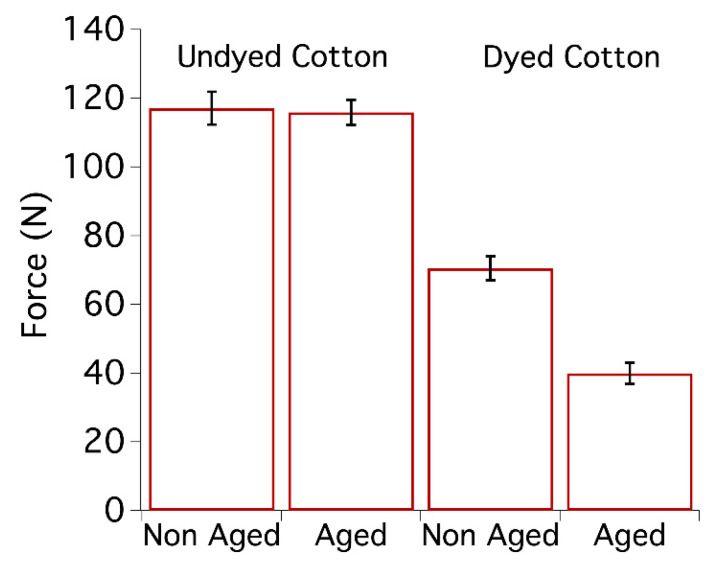
Maximum force at break of model cotton samples, undyed and dyed with iron-tannate dyes before [67] and after 10 years of natural ageing. From 5 to 10 tensile measurements per sample were carried out.

**Figure 2:**
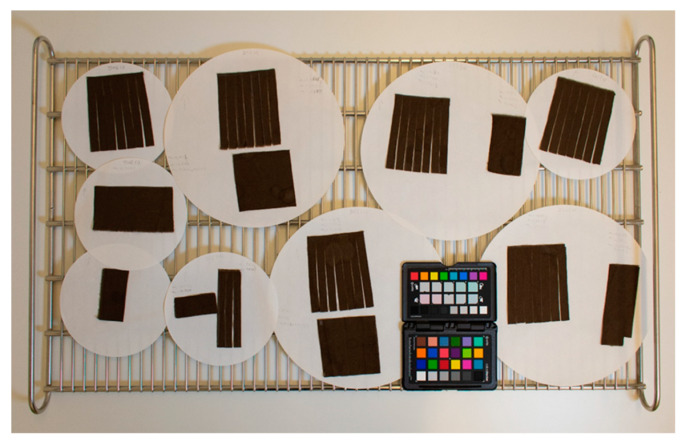
Cotton samples coloured with iron-tannate dyes, prepared for artificial ageing and tensile testing.

Cotton model textiles were prepared about 10 years ago using a Dye Jigger machine and an iron-tannate dye doped with copper (for further information about the dye composition and the preparation of samples, see reference [66]). The data related to the undyed and dyed cotton samples as prepared and after 10 years of natural ageing are reported in Figure 1. Dyed cotton samples prepared 10 years ago were used as reference systems and are labelled T0. As can be seen in Figure 2, the dyed cotton samples are dark black. The colourimetric coordinates of T0 before ageing are L^*^ = 37.0, a^*^ = 0.37, b^*^ = 1.41.

### 2.3. Sample Treatment

All the treatments were applied by nebulisation, except for treatment T8, which has the same composition of treatment T5 and was applied by brush. The rationale behind this choice is the following: as indicated below, the unique set of samples selected for this study is very fragile and cannot even withstand a spraying treatment. In general, it is expected that the application by brush delivers a higher amount of treatment with respect to spraying or nebulization; higher amount of treatment is also expected to provide an increase in the stabilization effect. In this sense, an application by brush may present a reference value to which treatments applied by nebulization, i.e., the only treatment deemed safe for these samples, can be compared. Due to the limited amount of samples, which were naturally aged for 10 years, we selected only one treatment to be applied both by nebulization and by brush, to be used to gather information about the effect in stabilization related to the different amount of the applied materials.

The nebuliser (INQUA Vernebler) was fed by an air compressor (COSTECH AS18B) operating at 2 bar. Nebulisation was given preference over spraying after preliminary tests because it was a gentler delivery method and would be a more realistic choice for treatments of already fragile textiles. Under these conditions, the samples were fully wetted during application. Samples were treated from both sides and sometimes using several application steps, where a minimum of 20 min drying time was allowed for between steps. The total amount of dispersion applied to each sample was calculated to deliver approximately 8 wt% of dry content relative to the weight of the sample. However, the real uptake was much lower; this is due to drift of material during nebulisation and less than 100% absorption capacities of the textiles’ samples. T0 refers to the untreated sample.

Below, details about the composition and the number of treatments applied on model samples are reported. The preparation of functionalised particles is described elsewhere [59,60,68].

Treatment 1—CMC@SNP + CNF (T1)

Treatment 1 consisted of a mixture of silica nanoparticles (SNP), functionalised with a cationic layer of polyethylenimine (PEI) and an anionic layer of carboxymethylcellulose (CMC) [from now on called CMC@SNP] and cellulose nanofibres (CNF). The mass ratio between CMC@SNP and CNF was 9:1. The weight concentration of the mixture was 1% in water/isopropanol mixture (1:1 by volume). Two applications were carried out; a total amount of about 8 mL was applied per gram of samples.

Treatment 2—PEI@SNP + CNF (T2)

Treatment 2 consisted of application of SNP, functionalised with PEI [from now on called PEI@SNP], followed by treatment with CNF. PEI@SNP was applied at a weight concentration of 1% water/isopropanol mixture (1:1 by volume), and CNF at 1% (water). Two applications were carried out; a total amount of about 8 mL of PEI@SNP and 4 mL of CNF was applied per gram of samples. A lower number of CNFs was applied with the aim of not hindering the penetration of PEI@SNP during the second application.

Treatment 3—PVP@SNP + CNC (T3)

Treatment 3 consisted of application of SNP, functionalised with a neutral layer of PVP [from now on called PVP@SNP], followed by treatment with cellulose nanocrystals (CNC). PVP@SNP was applied at a weight concentration of 1% water/isopropanol mixture (1:1 by volume), while CNC at 1.5% (water). Two applications were carried out; a total amount of about 8 mL of PVP@SNP and 4 mL of CNC was applied per gram of samples. A lower amount of CNC was applied with the aim of not hindering the penetration of PVP@SNP during the second application.

Treatment 4—CMC@CaCO_3_ (T4)

Treatment 4 consisted of application of calcium carbonate nanoparticles (CaCO_3_), functionalised with PEI and CMC [from now on called CMC@CaCO_3_]. CMC@CaCO_3_ was applied at a weight concentration of 2% (water/ethanol, 1:1 by volume). One application was carried out; a total amount of about 8 mL was applied per gram of samples.

Treatment 5—CMC@CaCO_3_ + PVP@SNP + CNC (T5)

Treatment 5 consisted of application of CMC@CaCO_3_, followed by treatment with PVP@SNP and with CNC. All the dispersions were applied at a weight concentration of 2%. One application was carried out; a total amount of about 8 mL of CMC@CaCO_3_, 8 mL of PVP@SNP and 1.7 mL of CNC was applied per gram of samples.

Treatment 6—CMC@CaCO_3_ + CNC (T6)

Treatment 6 consisted of application of CMC@CaCO_3_, followed by treatment with CNC. All the dispersions were applied at a weight concentration of 2%. One application was carried out; a total amount of about 8 mL of CMC@CaCO_3_ and 8 mL of CNC was applied per gram of samples.

Treatment 7—CMC@CaCO_3_ + CMC@SNP (T7)

Treatment 7 consisted of application of CMC@CaCO_3_, followed by treatment with CMC@SNP. CMC@CaCO_3_ was applied at a weight concentration of 2%, while CMC@SNP at 1%. One application was carried out; a total amount of about 8 mL of CMC@CaCO_3_ and 8 mL of CMC@SNP was applied per gram of samples.

Treatment 8—CMC@CaCO_3_ + PVP@SNP + CNC (T8)

Treatment 8 consisted of application of CMC@CaCO_3_, followed by treatment with PVP@SNP and with CNC. All the dispersions were applied at a weight concentration of 2%. One application by brush was carried out; a total amount of about 2 mL of CMC@CaCO_3_, 1.4 mL of PVP@SNP and 0.4 mL of CNC was applied per gram of samples.

### 2.4. Accelerated Ageing

To trigger the degradation of cellulose due to the presence of the iron-tannate dye, samples were aged at high temperature (70.5 ± 0.5 °C) and controlled relative humidity (49 ± 2%) in a WTC binder KBF240 oven. In the absence of a recognised standard for accelerated ageing of textiles in conservation research, 70 °C, rather than the more commonly used 80 °C, was chosen because the starting material was already severely degraded. 70 °C had also been used in related studies [67]. A constant RH typical for storage conditions was used to simulate as much as possible a realistic deterioration mechanism, which was proven to be more dependent on moisture than on temperature [69]. The ageing lasted for two weeks for the first set of samples (T1-3 + T0) and one week for the second set of samples (T4-8 + T0). Based on sample T0, whether ageing was conducted for one or for two weeks, degradation occurred to similar extent. Therefore, it was decided to use only one week in the second series of test.

### 2.5. Sample Characterisation

#### 2.5.1. Sample Conditioning and Weight Changes

After the treatment, the samples were left to dry in the fume hood and stored under controlled conditions (21 ± 1 °C and 50 ± 1% RH) before accelerated ageing. The aged samples were stored under the same conditions before the characterisation. The samples were weighed before and after the treatment to assess the uptake of the material.

#### 2.5.2. Tensile Testing

Tensile tests were used to evaluate the conservation status of the samples and the effectiveness of the applied treatment before and after the ageing. In a typical tensile test, samples are extended at a constant rate until rupture, recording the force over the increasing sample length (force-elongation curves). The first part of the curve is related to the response of the woven fabric to the application of the load; this behaviour is characterised by the elastic modulus at low deformation (EML [N/mm^2^]). Afterwards, due to the increasing stress, the final rupture of the textiles takes place, at the so-called maximum loading force at rupture [N]. The value of the elongation at break (E [%]) is a measure of the strength and flexibility of the samples.

Mechanical testing was performed using SHIMADZU Autograph AGS-X (Shimadzu, Kyoto, Japan) equipped with a static load cell of 100 N (for unaged and undyed samples a load cell of 10 kN was used). Before testing, the samples were conditioned at 21 ± 1 °C and 50 ± 1% RH. The measurements were carried out on warp direction of rectangular samples (1 × 10 cm^2^) at a constant extension rate of 10 mm/min and a gauge length of 50 mm [70]. From 5 to 10 repeats per sample were conducted. 

#### 2.5.3. Colourimetry

Reflectance spectra were acquired using a portable KONICA MINOLTA CM-2600D spectrophotometer (Konica Minolta, Tokyo, Japan) working in a λ range of 360–740 nm (with 10 nm resolution), equipped with an integrating sphere with a circular sampling spot (diameter 0.8 cm). Colourimetric coordinates were extracted from reflectance spectra using the standard illuminant D65 and a standard observer at 10°. Five points per sample were collected, each one with three averaged measurements. The CIE2000 formula [71] was used to calculate the difference ΔL^*^ before and after treatment and ageing. 

#### 2.5.4. SEM

SEM was performed with a JEOL JSM-IT500 (Jeol, Tokyo, Sweden) equipped with a tungsten filament and a Dry Silicon Drift detector. SMILE VIEW Lab was used for the data management. A small portion of each sample (0.25 mm^2^) was fasten on a stub, using carbon tape. To avoid sample charging, images were acquired at 5 kV with a fast frame-accumulation system. The working distance was set at around 10 mm, the probe current at 80 pA. When needed, a gold layer (about 6 nm thick) was deposited on samples with a rotary pump coater Q150R ES (Quorum Technologies, East Grinstead, UK).

#### 2.5.5. pH Measurements

pH measurements were performed to monitor the changes in the acidity of the samples before and after the ageing. A LAQUAtwin HORIBA pH meter (VWR, Radnor, PA, USA) working in the pH range 2.0–12.0 (resolution = 0.01), was used. Small samples (about 0.25 mm^2^) were weighed and covered with drops of water (about 1 mL/10 mg). After 16 h the pH of the extraction was measured. Measurements were performed in duplicate and the average results reported. The two measurements generally showed a variation of < 0.1; the highest variation noted was 0.4.

## 3. Results and Discussion

Iron-tannate inks or dyes are among the original components of cellulose-based artefacts and works of art that trigger severe degradation in cellulosic substrates. These water-based compounds have been widely used since ancient times as writing fluids or to colour woven and non-woven artefacts [66,72,73]. The colouring complex, iron (III) tannate, is obtained by reaction of tannic acids, from galls, leaves, or bark, with iron (II) sulphate. Sulfuric acid is generated along with the colouring complex and the strong acid catalyses the hydrolysis of cellulose. In addition, iron ions not involved in the formation of the colouring complex trigger further oxidation of cellulose [73]. Therefore, manuscripts written with iron gall inks or textiles coloured with iron-tannate dyes typically display severe degradation. Harsh dyeing conditions, including iron ions and sulphuric acid at high temperatures, are known to degrade textile fibres already during the dyeing process [66,74].

As shown in Figure 1, the maximum force at break of model cotton samples coloured with iron-tannate dyes, with a starting pH of about 3.5, was almost halved after 10 years of natural ageing. As a reference, an undyed model cotton sample (starting pH of about 6.5), aged under the same conditions, did not show any significant change in mechanical properties. In fact, for undyed systems, the difference falls within the experimental error calculated from repeated experiments.

The aim of the study being to evaluate the effect of a combined deacidification-strengthening treatment, cotton samples coloured with iron-tannate dyes (see Figure 2) were treated with either the strengthening only or the deacidification/strengthening formulations. The evaluation was conducted by assessing the mechanical properties with tensile testing, the fibre structure by SEM and the apparent colour changes by colourimetric measurements. pH was also measured to evaluate the buffering capacity of each treatment. The treated samples were then subjected to an accelerated ageing process and characterised using the same methodology.

The strengthening treatments capitalise on previously published work [55,59,60] and were based on silica nanoparticles (SNP) coated with CMC, polyethylenimine (PEI) or polyvinylpyrrolidone (PVP) combined with either cellulose nanofibres (CNF) or cellulose nanocrystals (CNC). The purpose of having CMC at the surface of the SNP was to increase the colloidal stability, which in turn improves the homogeneity of the deposition, while at the same time providing good interfacial compatibility with the cellulosic substrate. The surface coverage with PVP also enhanced the colloidal stability, while PEI was used to promote adhesion of the SNP to the negatively charged cellulosic surfaces [75]. The deacidification treatment consisted of nanoparticles (NP) of calcium carbonate surface modified with PEI and CMC in order to increase the colloidal stability, as previously discussed for silica nanoparticles [55,59,60].

### 3.1. Effect of the Nanosystems for Stabilisation Alone

Before testing the effect of a combined deacidification-strengthening treatment, the samples of iron-tannate dyed cotton were treated with the different strengthening formulations alone. Details about the treatments tested can be found in previously published work [55,59,60], where it was demonstrated that the combination of nanocellulose and SNP was efficient in terms of multiscale reinforcement, which is particularly important for textiles.

**Figure 3:**
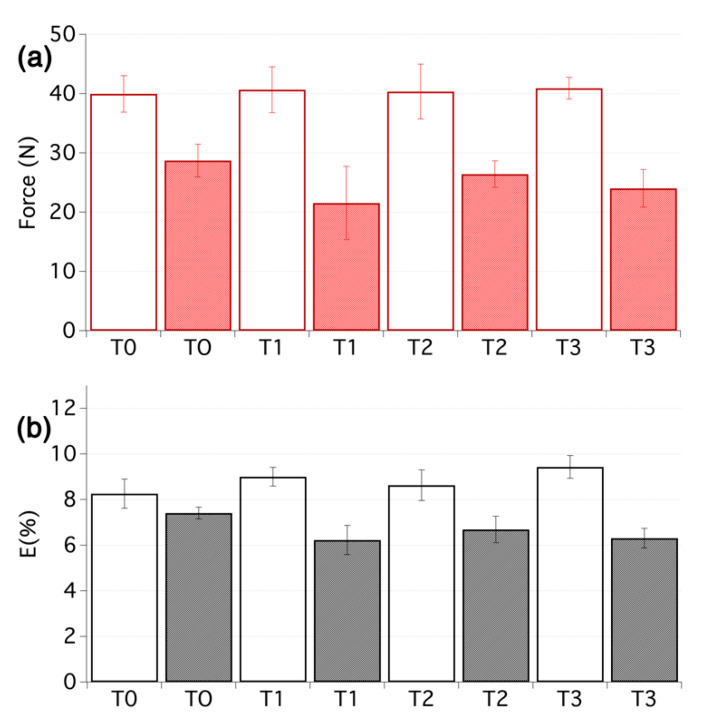
(**a**) Maximum force and (**b**) elongation at break for the first set of samples before ageing (empty bars) and after ageing (filled bars). From 5 to 10 tensile measurements per sample were carried out.

The effects of the different treatments on pH, mechanical properties and colour of the cotton samples are summarised in Table 1 and Figure 3 (samples T1–T3, T0 being the untreated reference sample).

When considering the results, it is important to bear in mind that the purpose of the treatments is to provide mechanical stabilisation over the ageing, and certainly not to provide a means to recover the original mechanical properties. Furthermore, it must be noted that these treatments only account for a total weight increase of 2.5 wt% or less. This should be compared to a weight increase up to 200%, which is common for traditional lining.

**Figure 4:**
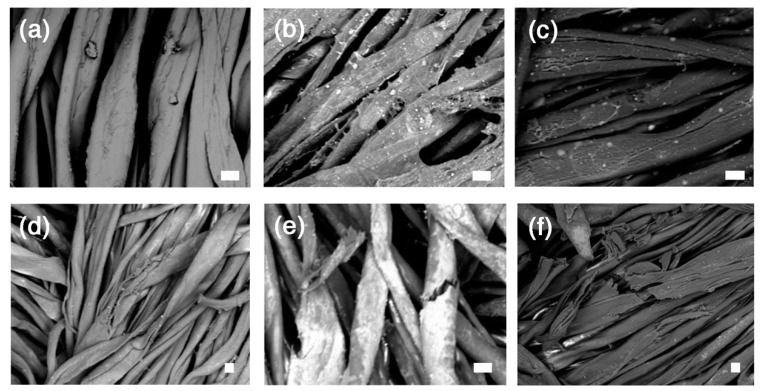
SEM images of samples belonging to the first set, before and after the artificial ageing. (**a**) T0 before ageing (1000× magnification); (**b**) T1 before ageing (950× magnification); (**c**) T3 before ageing (1000× magnification); (**d**) T0 after ageing (500× magnification); (**e**) T1 after ageing (1000× magnification); (**f**) T3 after ageing (500× magnification). The scale bar is 10 µm in all the images.

The first aspect of importance is the pH, as reported in Table 1. It is originally at 3.6. The treatments with SNP and nanocellulose raise this value to 4.3–4.4, most likely owing to the alkaline nature of silica, even though the variations observed are not far from the measurement error range. While the treatment of sample T1 induced a minor colour change, the lightness values were more affected for T2 and T3. It is difficult to come up with a plausible explanation for this difference, but surface aggregation of the SNP, which is somewhat supported by the SEM pictures (see Figure 4b,c) may be a contributing factor.

The effects of the treatment on the mechanical properties were moderate and the increase of the maximal force at break is within the experimental error, obtained from 5 to 10 replicas of tensile testing. In this sense, it is worth noting that on highly degraded materials such as those selected for the present study, a large set of samples must be tested to provide reliable mechanical data. The effect of the tested treatments was most noticeable for the elastic modulus at low deformation (EML), which consistently increased. This regime is of particular interest for textiles as it corresponds to the most common stress that these materials experienced. The effect can be attributed to the presence of nanocellulose that tends to act as a nanolining [55,60]. 

After artificial ageing, these positive effects were not retained and a dramatic loss of the mechanical properties was noted for all the samples, as one could have expected. As the textiles were not deacidified (see pH values after ageing in Table 1), the degradation continued and propagated towards the strengthening layers that are also cellulose-based. The EML was less affected, most likely because the improvement comes from the nanocellulose that was not so severely degraded during the time of the ageing, thanks to the high crystallinity of the material. The EML values are, however, prone to large error values, which must be kept in mind. 

Figure 4 shows SEM micrographs of samples after the strengthening. Owing to the application method and to the relatively low quantity deposited, the nanocellulose did not form a continuous film; instead, it mostly accumulated on the individual fibre surface without major inter-fibre bridging. This is in agreement with the observed enhancement of the mechanical properties. For samples T2 and T3 one can clearly notice some surface aggregates that could explain the changes in appearance of the samples. As shown by the mechanical data, the ageing clearly induced further damage of the samples, also on the treated samples (see the SEM images of Figure 4d–f). On the basis of the experimental results shown here, we decided to test a combined treatment featuring alkaline nanoparticles and stabilizing agents on a new set of samples. Even if both CNC and CNF performed poorly on dyed textiles in the absence of a deacidification treatment, we previously demonstrated that both systems are effective for canvas stabilization and that CNC performs better than CNF [55]. In fact, CNC presents practical advantages such as the possibility to use higher solids content in the aqueous dispersion to be applied, allowing for higher reinforcement per equivalent number of coatings and lower mechanical changes upon RH variations. Moreover, CNC has a higher degree of crystallinity compared to CNF, which may be a key towards the stability of the treatment in a buffered, i.e., less degrading, environment. Therefore, for the second set of samples, we decided to focus on CNC and SNP only to be used in combination with alkaline nanoparticles.

### 3.2. Effect of Combined Nanosystems for the Strengthening and Deacidification

Samples T5-T8 were treated first with calcium carbonate nanoparticles for deacidification, and then with strengthening materials similar to the ones used in 3.1. The first noticeable difference is the pH of the samples, which was raised above 7 for all the treatments conducted (see Table 1). While the presence of CaCO_3_ NP was beneficial for the buffering of the samples, it had a bigger impact on the colour of the sample than the strengthening treatment alone, as noted by a large loss in L-value. It is noteworthy that for these systems, the pick-up is about 5 wt%, except for samples T4, which was treated with the sole alkaline nanoparticles, and T8 that has the same composition of T5 except for the fact that the treatment was applied by brush. It is worth recalling that, as indicated in the experimental section, it is expected that the application by brush delivers a higher amount of treatment with respect to nebulization, with a consequent increase in the stabilization effect. In this sense, an application by brush presents a reference value, which treatments applied by nebulization, i.e., the only treatment deemed safe for the unique set of samples used in this study, should be compared to.

From a mechanical point of view, the reinforcement was a bit stronger, especially for samples T7 and T8, which were treated with materials that were previously proven to be effective in addressing severely degraded cotton [59,60], even if here the increase is less than expected, probably owing to the poor starting condition of the textiles, which is also probably responsible for the slightly high dispersion of maximum force at break values, as shown, for instance, by sample T7.

**Figure 5:**
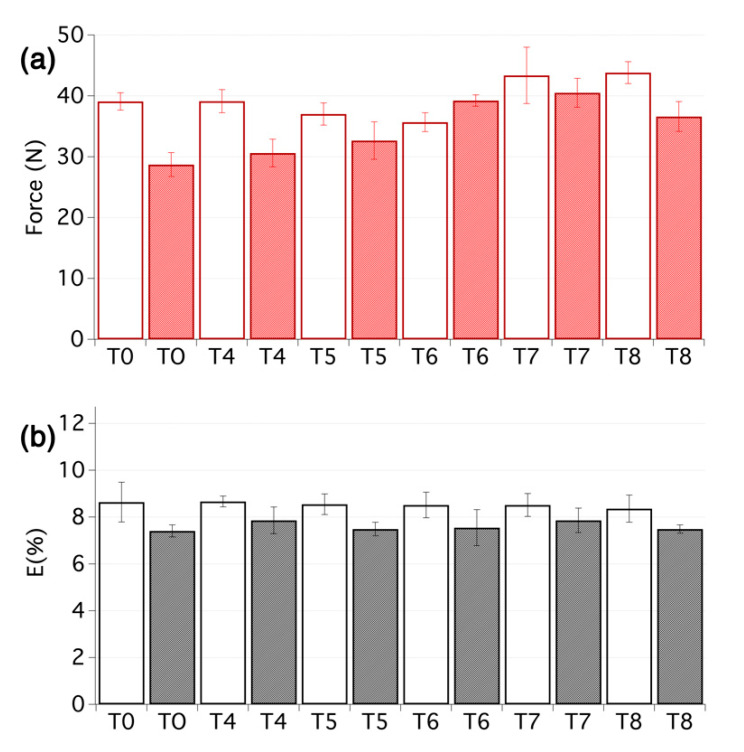
(**a**) Maximum force and (**b**) elongation at break for the second set of samples before ageing (empty bars) and after ageing (filled bars). From 5 to 10 tensile measurements per sample were carried out.

Artificial ageing was conducted using similar conditions of temperature and humidity, as discussed above. The buffering capacity of the deacidification treatment could clearly be seen and the pH values of the aged samples remained close to the values obtained right after the treatment (see Table 1, T4–T8). This, in turn, had important consequences for the stability of the treatment. In fact, all the treated samples showed increased resistance to ageing, compared with the untreated textiles, especially in terms of maximum force at break, as shown in Figure 5. In particular, samples that had undergone the combined deacidification-strengthening procedure display a considerable increase ranging from 14% (T5) to 40% (T7), which are quite high values considering the small weight increase due to the treatments, as reported in Table 1. In this sense, it is worth noting that treatment T6 and T7 provided a slightly better stabilization effect than treatment T8, which as above indicated, being applied by brush, can be used as a reference value to which the nebulized treatments, which are safer to fragile artefacts and delivered about 30% less material over the treated surfaces, should be compared to. It is also worth noting that the elongation at break remained close to the original values, right after the treatment, which is in strong contrast with the samples that were not deacidified. It clearly underlines that the deacidification not only affects the substrate but also the strengthening itself. The EML values tended to increase due to all treatments, without a clear difference in trends between the samples with or without deacidification, and were generally even higher after accelerated ageing. This is probably due to interlocking of the fibres and of the yarns and threads, which in turn renders the textiles stiffer. 

**Figure 6:**
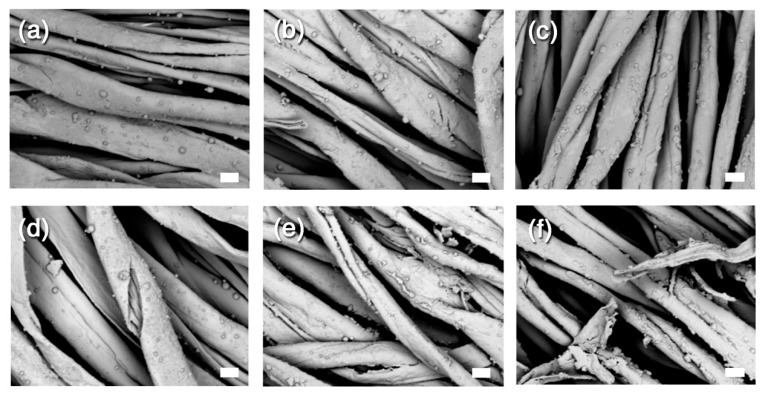
SEM images of samples belonging to the second set of samples, before and after the artificial ageing. (**a**) T4 before ageing; (**b**) T5 before ageing; (**c**) T7 before ageing; (**d**) T4 after ageing; (**e**) T5 after ageing; (**f**) T7 after ageing. The scale bar is 10 µm in all the images (1000× magnification).

In agreement with these results, the SEM pictures displayed in Figure 6 showed much less difference before and after ageing, especially in terms of crack formation. Interestingly, one can clearly see the presence of micron-sized clusters of CaCO_3_ NPs (whose diameter is around 90 nm). The ΔL^*^ values measured on the second series of samples are higher than those measured on the first set of samples. The difference is probably due to the highest amount of treatment applied on each sample, as reported in Table 1, which leads to a darkening of the surface (negative ΔL^*^ values). However, it is worth noting that ageing of iron-tannate cotton leads to an increase in redness and yellowing of the samples, which was attributed to the breakdown of the blue-black iron-tannate dye complex [66]. In this sense, an increase in the darkness of the samples as a result of treatment and ageing can partially mitigate the colour changes due to the breakdown of the original colouring complex.

## 4. Conclusions

Given its ubiquitous presence, the degradation of fibre-based cellulosic materials is a central topic in conservation science. The use of nanomaterials for conservation of artefacts made from such materials seems to be a promising approach, allowing milder and more controlled treatments than what is commonly used today. Since acid-catalysed hydrolysis is an important mechanism behind the degradation, it is important to include deacidification in the conservation procedure. A combined deacidification-strengthening approach is, therefore, an appealing way forward.

Here, taking the case of iron-tannate dyes, we studied the possibility of using silica nanoparticles combined with nanocellulose to stabilise a unique set of naturally aged (10 years) model samples, with and without a deacidification treatment by calcium carbonate nanoparticles. Such a combined treatment seems not to have been reported in the literature before.

The treatment by the strengthening agents provided stabilisation of the cotton, giving a slight increase of the mechanical properties at a weight increase around 2.5 wt%. When subjected to accelerated ageing, the loss of mechanical properties, evaluated by tensile testing measurements, continued, as the intrinsic textiles acidity further degraded the original substrate. The acidity generated probably also induced degradation of the reinforcing agents, which were in part cellulose-based.

The situation was very different when deacidification was conducted prior to application of the strengthening formulation. The pH changed from ~3.6 to ~7.5 and the gain in the mechanical properties was higher, especially with respect to the maximum force at break. After artificial ageing, the effect remained and the CaCO_3_ NPs continuously buffered the material, slowing down the degradation of the cotton. The alkaline nanoparticles also acted as a protecting agent for the materials used for the strengthening. 

These results demonstrate the potential of using a combined deacidification-strengthening approach in the stabilisation of degraded fibrous cellulosic materials. They also demonstrate the need for a better understanding of the cross interaction of these systems and to which extent the adhesion of the nanomaterial to the treated surface affects the reinforcement. 

In our view, these results lay the ground for further development of complex formulations based on the combination of nanomaterials that are environmentally benign, easy to handle and have the potential to be adopted in standard conservation practice.

## Figures and Tables

**Table 1:** Effect of the different treatments before and after the artificial ageing on pH, elastic modulus at low deformation (EML) and colour change in terms of lightness change (ΔL^*^). Weight uptakes are also reported.

System	Weight Uptake (%)	Before Ageing			After Ageing			Action Provided by the Systems
		pH	EML (N/mm^2^)	ΔL^*^	pH	EML (N/mm^2^)	ΔL^*^	
T0	-	3.6	214 ± 64	-	4.1	253 ± 2	−1.8	N/A
T1	2.0	4.3	287 ± 27	−1.6	4.4	309 ± 8	−3.7	Strengthening
T2	1.5	4.3	333 ± 66	−4.1	4.2	317 ±24	−7.5	Strengthening
T3	2.5	4.4	234 ± 36	−5.2	4.4	359 ±79	−6.3	Strengthening
T0	-	3.7	181 ± 32	-	4.5	310 ±25	−1.6	N/A
T4	2.5	7.3	192 ± 25	−7.3	7.6	255 ± 43	−9.4	Deacidification
T5	4.9	7.4	290 ± 45	−9.0	7.5	305 ± 34	−8.6	Deacidification/Strengthening
T6	4.7	7.5	250 ± 51	−9.1	7.3	358 ± 94	−8.4	Deacidification/Strengthening
T7	4.7	7	195 ± 54	−13.6	7.1	383 ± 43	−12.6	Deacidification/Strengthening
T8	7.3	7.4	336 ± 82	−9.4	7.4	443 ± 22	−9.3	Deacidification/Strengthening

## Abbreviations

CMC	carboxymethyl cellulose
CNC	cellulose nanocrystals
CNF	cellulose nanofibres
DP	degree of polymerisation
EML	elastic modulus at low deformation
NMR	nuclear magnetic resonance
NP	nanoparticle
PEI	polyethylenimine
PVP	polyvinylpyrrolidone
RH	relative humidity
SEM	scanning electron microscopy
SNP	silica nanoparticle
T	temperature
